# Resistance patterns among drug-resistant tuberculosis patients and trends-over-time analysis of national surveillance data in Gabon, Central Africa

**DOI:** 10.1007/s15010-022-01941-5

**Published:** 2022-10-28

**Authors:** Jabar Babatunde Pacome Achimi Agbo Abdul, Bayode Romeo Adegbite, Micheska Epola Dibamba Ndanga, Jean Ronald Edoa, Rhett Chester Mevyann, Guy Rogue Arnault Ibinda Mfoumbi, Tshisekedi Jean de Dieu, Jocelyn Mahoumbou, Christopher Mebiame Biyogho, Sankarganesh Jeyaraj, Stefan Niemann, Bertrand Lell, Peter Gottfried Kremsner, Abraham Sunday Alabi, Ayola Akim Adegnika, Martin Peter Grobusch

**Affiliations:** 1 Centre de Recherches Médicales de Lambaréné CERMEL, Hospital Albert Schweitzer, BP 242, Lambaréné, Gabon; 2 Center of Tropical Medicine and Travel Medicine, Department of Infectious Diseases, Amsterdam University Medical Centers, Location Amsterdam, Amsterdam Infection and Immunity, Amsterdam Public Health, University of Amsterdam, Meibergdreef 9, 1105 AZ Amsterdam, The Netherlands; 3 Institut für Tropenmedizin, Eberhard Karls Universität Tübingen and German Center for Infection Research (DZIF), Tübingen, Germany; 4 Programme National de Lutte Contre la Tuberculose, Libreville, Gabon; 5 PSG Center for Molecular Medicine and Therapeutics, PSG Institute of Medical Sciences and Research, Coimbatore, India; 6 National Reference Center for Mycobacteria, Forschungszentrum Borstel, Borstel, Germany; 7 Health Focus GmbH, Potsdam, Germany; 8 German Center for Infection Research, Tübingen, Germany; 9 Department of Parasitology, Leiden University Medical Center, Leiden, The Netherlands; 10 Masanga Medical Research Unit, Masanga, Sierra Leone; 11 Institute of Infectious Diseases and Molecular Medicine, University of Cape Town, Cape Town, South Africa

**Keywords:** Rifampicin resistance, Drug resistance, MDR-TB, XDR-TB, pre-XDR-TB, Lambaréné, Gabon

## Abstract

**Objective:**

Routinely generated surveillance data are important for monitoring the effectiveness of MDR-TB control strategies. Incidence of rifampicin-resistant tuberculosis (RR-TB) is a key indicator for monitoring MDR-TB.

**Methods:**

In a longitudinal nationwide retrospective study, 8 years (2014–2021) of sputum samples from presumptively drug-resistant tuberculosis patients from all regions of Gabon were referred to the national tuberculosis reference laboratory. Samples were analysed using GeneXpert MTB/RIF and Genotype MTBDRsl version 2/Line Probe Assay.

**Results:**

Of 3057 sputum samples from presumptive tuberculosis patients, both from local hospital and from referral patients, 334 were RR-TB. The median patient age was 33 years (interquartile range 26–43); one third was newly diagnosed drug-resistant tuberculosis patients; one-third was HIV-positive. The proportion of men with RR-TB was significantly higher than that of women (55% vs 45%; *p* < 0.0001). Patients aged 25–35 years were most affected (32%; 108/334). The cumulative incidence of RR-TB was 17 (95% CI 15–19)/100,000 population over 8 years. The highest incidences were observed in 2020 and 2021. A total of 281 samples were analysed for second-line drug resistance. The proportions of study participants with MDR-TB, pre-XDR-TB and XDR-TB were 90.7% (255/281), 9% (25/281) and 0.3% (1/281), respectively. The most-common mutations in fluoroquinolones resistance isolates was gyrA double mutation gyrA MUT3B and MUT3C (23%; 4/17). Most (64%; 6/8) second-line injectable drugs resistance isolates were characterised by missing both rrs WT2 and MUT2 banding.

**Conclusion:**

The increasing incidence of MDR-TB infection in Gabon is alarming. It is highest in the 25–35 years age category. The incidence of MDR-TB infection in treatment-naïve patients calls for case finding and contact tracing strategy improvement.

**Supplementary Information:**

The online version contains supplementary material available at 10.1007/s15010-022-01941-5.

## Introduction

Multidrug-resistant tuberculosis (MDR-TB) is defined as tuberculosis caused by mycobacteria being resistant to at least rifampicin and isoniazid (the two essential first-line therapy backbone compounds) [1]. The global threat of MDR-TB highlights the need of identifying such resistance as soon as possible. About 470,000 people were diagnosed with MDR-TB and approximately 180,000 die every year, according to recent World Health Organization (WHO) estimates [1]. Rifampicin-resistant tuberculosis (RR-TB) infection is caused by a strain that is resistant to rifampicin with or without resistance to other first-line anti-TB drugs. Studies on drug resistance development show that isoniazid resistance develops first in most cases, followed by resistance to rifampicin or ethambutol, then resistance to pyrazinamide, and lastly, resistance to second- and third-line drugs [2–4]. Therefore, RR-TB without resistance to second-line drugs is generally considered as amounting to MDR-TB [4].

Gabon is one of the high-burden tuberculosis countries with an incidence of 527 per 100,000 population [5, 6], with a high prevalence of multidrug-resistant tuberculosis (MDR-TB) [7]. The alarming situation of the emergence of MDR-TB triggered a major effort to establish molecular surveillance and clinical care capacity in the country [7, 8].

Few Sub-Saharan African countries report data on the burden of DR-TB based on periodic national data assessments [9, 10]. We describe the national trends and the resistance pattern of rifampicin-resistant TB over 8 years (2014–2021) in Gabon.

## Methods

### Study design and participants

This is a retrospective longitudinal study using the national drug-resistant tuberculosis data. All records of patients with drug-resistant tuberculosis whose samples were submitted during the period between January 2014 and December 2021 were eligible. The STrengthening Reporting of Observational Studies in Epidemiology (STROBE) guideline has been followed to report this study [11].

### Study setting and data collection

The national tuberculosis control programme (PNLT) extended the national rifampicin resistance diagnostic capacity by providing each province with a GeneXpert machine in 2018. All of the provinces ship the rifampicin-resistant samples to the TB Reference Laboratory of the Centre de Recherches Médicales de Lambaréné (CERMEL), Gabon. As recommended by Gabon’s national TB guidelines, the following patients are eligible for GeneXpert testing: children younger than 15 years; people living with HIV; patients with a history of TB treatment; case contacts of MDR-TB patients; and any patients considered to be at risk of having RR-TB by the attending physician. In case RR-TB is detected, second-line anti-TB drug resistance testing was performed. Since data were collected before the WHO 2021 updated definition of XDR and pre-XDR tuberculosis, the cases were defined and treated following the 2013 consolidated guidelines on drug-resistant tuberculosis [12]. Therefore, rifampicin resistance without resistance to the second-line drugs (levofloxacin, moxifloxacin, kanamycin, amikacin and capreomycin) was considered to be MDR-TB and treated with second-line anti-TB drugs. Pre-extensively, drug-resistant tuberculosis (pre-XDR-TB) was defined as resistance to at least rifampicin plus either a fluoroquinolone (levofloxacin or moxifloxacin) or a second-line injectable anti-TB drug (kanamycin, amikacin or capreomycin), while extensively drug resistance tuberculosis (XDR-TB) as resistance to at least rifampicin to a fluoroquinolone and to a second-line injectable anti-TB drug.

### Sputum sample collection and laboratory analysis

Two sputum samples from patients with presumptive pulmonary tuberculosis were collected. The sputa of patients with rifampicin resistance tuberculosis detected by GeneXpert MTB/RIF (Cepheid, Sunnyvale, CA, USA) were further analysed for second-line drug resistance using the Genotype MTBDRsl/Line probe assay (Hain Life Science GmbH, Nehren, Germany). The GeneXpert MTB/RIF and Genotype MTBDRsl/Line probe assay tests were carried out according to the manufacturer’s instructions [13, 14]. The line probe assay is a deoxyribonucleic acid (DNA) strip-based test determining *Mycobacterium tuberculosis* drug-resistant strains. It identifies resistance due to mutations in gyrA (from codon 85–96) and gyrB (from codon 536–541) [2] genes for fluoroquinolone (levofloxacin or moxifloxacin), the rrs gene’s (nucleic acid position 1401, 1402 and 1484) and the eis gene’s promoter region (from − 37 to − 2 nucleotides upstream) for detection of resistance to the second-line injectable anti-TB drugs (kanamycin, amikacin or capreomycin) used during the earlier years of the study period. Mutations are detected by either the binding of amplicons to probes targeting the most commonly occurring mutations (MUT probes); or by the lack of hybridisation (i.e. lack of binding) of the amplicons to the corresponding WT probes [13].

### Data management and statistical analysis

The cumulative incidences with confidence intervals per (CI) 100,000 were calculated using RR-TB cases per the population estimate based on data from the National Population Census and Worldometer estimation [15]. Statistical analyses were performed using RStudio (R Foundation for Statistical Computing, Vienna, Austria). Quantum Geographic Information System software was used to identify the spatial trends of RR-TB and GeneXpert machine availability [16]. We investigated the variations in trends of RR-TB, MDR-TB and pre-XDR-TB incidences and estimated the average annual per cent change (AAPC) with their 95% CI by means of a Joinpoint Trend Analysis software (Version 4.9.1.0, USA) [17].

### Ethical consideration

Use of routine retrospective data was approved by the Institutional Ethics Committee of CERMEL (CEI/0152021). No additional data were collected from patient.

## Results

### Characteristics of patients with RR‑TB

**Fig. 1:**
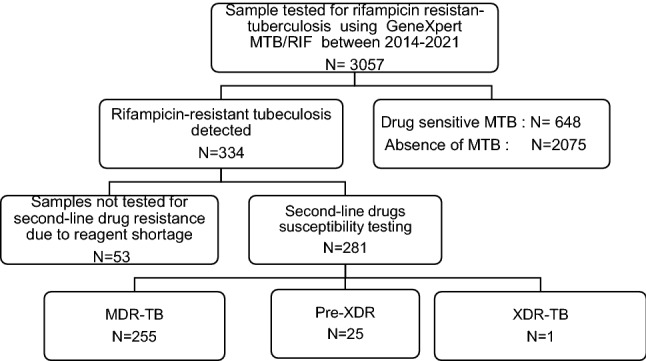
Flow chart of patient recruitment and drug-resistant tuberculosis pattern in Gabon between 2014 and 2021. MDR-TB( muti-drug resistant tuberculosis ): resistance to rifampicin and resistance to isoniazid, without resistance to any second-line drugs; pre-XDR-TB( pre-extensively drug-resistant tuberculosis): resistance to at least rifampicin plus either a fluoroquinolone ( levofloxacin, or moxifloxacin) or a second-line injectable anti-TB drug (kanamycin, amikacin, or capreomycin); XDR-TB (Extensive drug resistant tuberculosis): resistance to any fluoroquinolone and to at least one of three second-line injectable drugs (capreomycin, kanamycin and amikacin), in addition to multidrug resistance

Samples analysed included all samples received during the study period, including samples from the local hospitals, as well as referral samples. Only rifampicin-resistant samples were referred to CERMEL. Rifampicin-sensitive samples from other sites were not included in this study; as this study focused on drug resistance surveillance data only, we were unable to provide data for drug-sensitive cases. Among a total of 3057 presumptive tuberculosis patients sputum samples analysed during the study period, 334 rifampicin-resistant TB patients were identified (Fig. 1). The median age of patients with RR-TB was 33 years (IQR 26–43). A total of 103/334 (31%) RR-TB patients were newly diagnosed with tuberculosis, and the HIV-positive proportion was 35% (118/334) (Table 1). The HIV infection was more prevalent in 35–45 years and 46–55 years categories (Supplementary File S1).

**Table 1:** Socio-demographic and baseline clinical characteristics of patients with rifampicin-resistant tuberculosis, Gabon, 2014–2021

Characteristic	All patients *N* = *334 (%)*	*p* value
Age group (years)		** < 0.0001**
15–24	55 (17)	
25–34	108 (32)	
35–44	67 (20)	
45–54	65 (19)	
55 and more	39 (12)	
Sex		** < 0.0001**
Female	150 (45)	
Male	184 (55)	
HIV status		** < 0.0001**
Negative	189 (57)	
Positive	118 (35)	
Unknown	27 (8)	
Patient category		** < 0.0001**
New patient	103 (31)	
Previously treated for TB	174 (52)	
Unknown	57 (17)	

Statistically significant *p* values are in bold

### Incidence rate of rifampicin-resistant tuberculosis

**Fig. 2:**
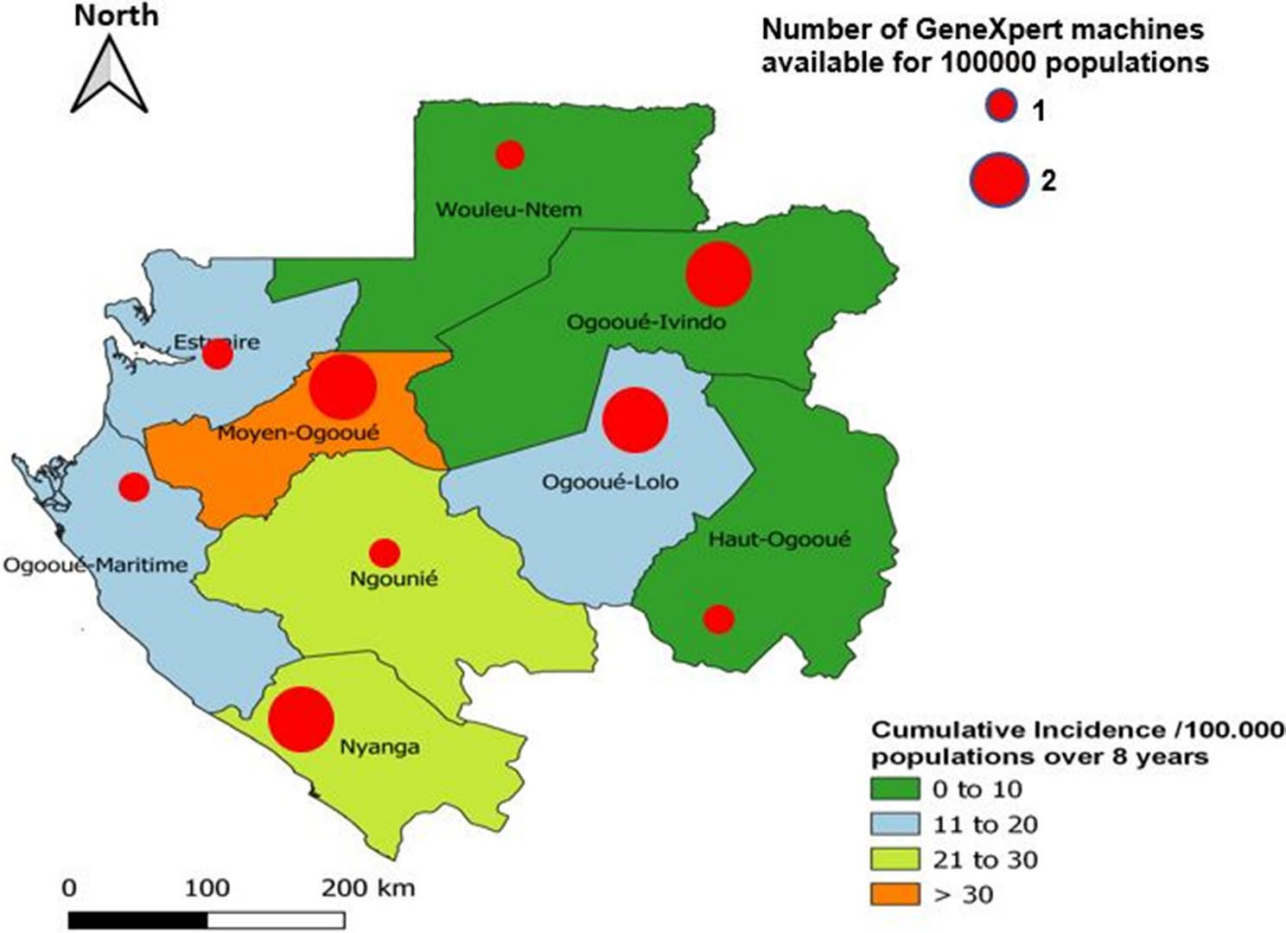
Rifampicin-resistant tuberculosis incidence per 100,000 population, Gabon, between 2014 and 2021, superposed with GeneXpert machine availability

Overall, the cumulative incidence of RR-TB was 17(95% CI 15–19)/100,000 inhabitants over 8 years. More than half (54%; 181/334) of the cases were from Estuaire Province (the province around Libreville, the capital with an estimated 638,220 inhabitants in 2021); 26% (86/334) cases were from Moyen-Ogooué Province (the province holding the reference tuberculosis laboratory), 7% (24/334) were from Ngounié, 6% (19/334) from Ogooué Maritime, 5% (16/334) from Woleu-Ntem, and 2% (8/334) from Nyanga and Ogooué-Lolo provinces, respectively. The cumulative incidence of TB per province (region) showed that the Moyen-Ogooué had the highest incidence of RR-TB (Fig. 2). Patients aged between 25 and 34 years were most affected (32%, 108/334); the proportion of men with RR-TB was significantly higher than that of women (55% vs 45%; *p* < 0.0001; Table1).

### Drug resistance patterns

A total of 53 out of 334 samples were not passed on for second-line drug resistance analysis because of temporary reagent shortage. The proportion of study participants having pre-XDR-TB and XDR-TB was 9% (25/281) and 0.3% (1/281), respectively (Fig. 1). The proportion of patients with MDR-TB was 90.7% (255/281). A total of 17 fluoroquinolones resistance isolates were identified. The most common fluoroquinolone resistance banding pattern observed was the gyrA double mutation gyrA MUT3B and MUT3C (23%); 41% of isolates showed missing gyrA WT3 and MUT3A, MUT3B, MUT3C and MUT3D banding. For second-line resistance, eight defined mutations in the *rrs* gene were detected, and most (64% 6/8) of the isolates were characterised by missing of *rrs* WT2 and MUT2 banding. The XDR-TB case isolate was characterised by missing of both *gyrA* WT3 and MUT3A, MUT3B, MUT3C, MUT3D banding and *rrs* WT2 and MUT2 banding (Table 2).

**Table 2:** Polymorphism patterns detected by genotype MTBDRsl assay in drug‑resistant *Mycobacterium tuberculosis* strains

Second-line drugs	Genes	MTBDRs probe			Clinical implications
Fluoroquinolones resistance	*gyrA gene*	MUT 3A		2 (12)	Levofloxacin is not effective Moxifloxacin could be used at higher dose
		MUT3B		2 (12)	Levofloxacin is not effective Moxifloxacin is not effective
		MUT 3C		1 (6)	
		MUT3D		1 (6)	
		MUT3B and MUT3C		4(23)	
		gyrA WT3, MUT3A, MUT3B, MUT3C, MUT3D not developed		8 (41)	Levofloxacin is not effective Moxifloxacin could be used at higher dose
Total				*N* = 17(100%)	
Second-line injectable drugs resistance	rrs gene	MUT 1		1 (12)	Amikacin, kanamycin and capreomycin are not effective
		rrs WT1 and MUT1 not developed		1 (12)	Kanamycin and capreomycin are likely not effective
		MUT 2	1 (12)		Amikacin, kanamycin and capreomycin are not effective
		rrs WT2 and MUT2 not developed	6 (64)		Kanamycin and capreomycin are likely not effective
Total			*N* = 8(100%)		

### Trends in incidence of rifampicin-resistant, pre-XDR and XDR-TB in Gabon 2014–2021

**Fig. 3:**
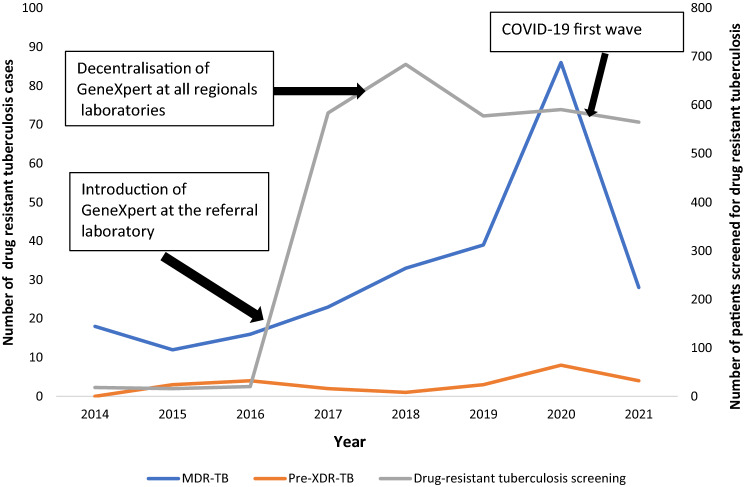
Trends over time of specimen sent in, and testing positive, for rifampicin-resistant tuberculosis in Gabon’s referral tuberculosis laboratory between 2014 and 2021

Figure 3 shows the trends in drug-resistant tuberculosis screening and its incidence from 2014 to 2021. The screening for drug-resistant tuberculosis increased significantly from 2016 to 2021, with the highest numbers observed between 2018 and 2020, before decreasing between 2020 and 2021. The incidence of RR-TB increased over time from one single case in 2014 to four cases per 100,000 inhabitants in 2021. The Joinpoint analysis showed that the incidence of RR-TB and MDR-TB has been increasing significantly by 18.7% per year (95% CI 2.9–37.1; *p* = 0.027) and 18.8% (95% CI 0.8–39.8; *p* = 0.042). The incidence of pre-XDR-TB did not change significantly (AAPC: 8.1; 95% CI − 37.5 to 87.2; *p* = 0.78).

## Discussion

We report pattern and trends over an 8-year time period of multidrug-resistant tuberculosis in Gabon. The overall incidence of MDR-TB is higher than what was reported from Uganda, the Republic of Congo [18] and Botswana [19], but lower than what was reported from South Africa in 2019 [20]. The higher burden of MDR-TB could be explained by the lack of resources to properly implement and supervise first-line drug treatment and might be owed in part to drug stock-outs. Indeed, the TB treatment in Gabon is challenged by the intermittent unavailability of first-line drugs treatment leading to involuntary discontinuation of TB treatment by the patients [21]. A contributor to the higher rate of loss-to-follow-up and discontinuation of treatment [7, 22] is due to the fact that most of the patients in the rural areas of the country live far from diagnostic and treatment facilities. This makes the directly observed TB treatment difficult to be applied. Our results further show that MDR-TB increased other time, with the highest incidence observed between 2019 and 2020. It could be explained by the fact that between 2017 and 2018 there was an unusual long period of first-line drug shortages and drugs were intermittently provided to patients. Moreover, in 2018, with the support of the Global Fund, the national tuberculosis control programme initiated the de-centralising of using GeneXpert in patients at risk of RR-TB. All of this contributed to the highest RR-TB case detection rates during 2018 and 2020 period. This case-finding strategy dynamics have been disrupted significantly by the COVID-19 pandemic which consequently reduced the number of RR-TB patients screened between 2020 and 2021 (Fig. 3).

In our study, RR-TB was most frequently observed in 25- to 35-year-old individuals. This is in line with the baseline characteristics of TB in Gabon reported earlier [8, 22]. Our findings are comparable with what was reported by other studies conducted in Africa [9, 23, 24]. This population category is the most economically and socially active, with many contacts beyond the own household, being most exposed to, and contributing most to the spread of tuberculosis. Furthermore, several tuberculosis-related co-morbidities including HIV infection, diabetes, smoking, illicit drug abuse, harmful alcohol consumption and mental health issues begin or worsen within this age category [25]. Finally, they encounter challenges in having access to adequate care due to many socio-economic factors. Therefore, tuberculosis control action should be intensified among adolescents and young adults in settings like those in Gabon. The high proportion of naïve TB treatment patients diagnosed with MDR-TB in the present national surveillance data-based study is an additional reason for adjusting and intensifying case finding and earlier initiation of MDR-TB treatment. Interestingly, the Moyen-Ogooué Province yielded 26% of cases and the highest cumulative incidence per 100,000 inhabitants. This could be explained by the fact that the region holds the referral laboratory and the research centre where patients are actively screened for tuberculosis. The highest incidence is explained by the relatively small size of the population of this region as compared with the Estuaire Province where more than the half of the case was diagnosed.

Compared with other Central African countries (Congo and Cameroon), the proportion of pre-XDR TB in our study is higher [18, 26], but lower as compared to what was reported from South Africa [20]. The studies in Congo and Cameroon were not conducted at national level; however, the WHO country report confirms that the burden of MDR and pre-XDR TB in Gabon is the highest among those countries [27]. However, the small size of Gabon’s general population should be taken into account for interpretation. The higher number of pre-XDR TB in Gabon could also be explained by the incorrect and/or overuse of fluoroquinolones by some clinicians prior to the implementation of the national TB-MDR guidelines. The predominance of pre-XDR-TB has a number of important implications. Firstly, all patients with RR-TB (detected by GeneXpert) should mandatorily have also fluoroquinolones and other second-line anti-TB drugs tested for resistance before initiation of second-line TB treatment. Secondly, clinicians and decision-makers should safeguard that the first-line drug-sensitive TB treatment guideline is followed properly to reduce the increasing of MDR-TB.

The trend of pre-XDR incidence did not change significantly from 2014 to 2021. This is due to the fact that since the first implementation in Gabon of the short MDR-TB treatment regimen and its recommendation by our team at national level [8] up to 2020 (before the update of national MDR-TB treatment following the updated WHO recommendation replacing injectable second-line drugs), the MDR-TB patients were hospitalised during the whole treatment period and those receiving injectable drug ambulatorily, taking the oral drug under supervision. The patients are managed in selected health care centres in the country. This allows for rational use of the second-line drug and patient treatment compliance. However, the treatment is now increasingly becoming decentralised, including a range of regional hospitals to reduce patient transfer. The first case of XDR-TB was observed only recently (October 2021). The male patient was 38 years old, non-smoking, HIV-negative, and treated successfully (based on repeated smear negativity and ‘clinical cure’) in 2012 for drug-sensitive tuberculosis. This first case of XDR-TB alerted health authorities to the need of patients and health care training as well as an improved tuberculosis infection control strategy in general to avoid spreading of XDR *Mycobacterium tuberculosis* in the community.

As reported previously, our study confirms that most fluoroquinolone resistance is conveyed by gyrA mutations [28]. The detection of gyrase mutations helps to predict the presence and level of fluoroquinolone resistance. The high proportion of strains showing absences of both wild type and polymorphisms in our study is an additional indication of the value of having the phenotypic drug sensitivity test (DST) and strain sequencing capacity in the referral tuberculosis laboratory to improve patient treatment, by adapting the drug regimen to the *Mycobacterium tuberculosis* strain causing the individual infection. To uncover transmission patterns at the population level, proper public health action based on tailored molecular-guided cluster analysis is ongoing by our research team. The sputum of patients is prospectively collected for the sequencing of the strain. The samples are sent to the collaborating centre in Borstel, and training of local staff is done for capacity development.

Our study is subject to the usual limitations inherent to retrospective routinely generated laboratory surveillance data. Moreover, there were some cases of RR-TB cases that did not undergo second-line DST, as no sample was sent to the national referral TB laboratory. We were not able to perform the phenotypic drug sensitivity test. The true national incidence of RR-TB might be slightly different from what is reported in our study. However, we received samples from patients living in all of the nine provinces of the country; therefore, our findings most likely provide a fair reflection of the general trends in RR-TB incidence in Gabon over the study period, assisting policymakers to adjust the MDR-TB control strategy. Given the rising prevalence of pre-XDR-TB surveillance monitoring of TB treatment should be improved. We cannot exclusively associate the high incidence with an increase in cases. During the study period, there was an improvement in the case finding strategy and diagnostic capacity of each regional laboratory. Consequently, an updated study will be necessary in future in order to confirm the hypothesis of a surge of cases of drug-resistant tuberculosis in Gabon.

## Conclusion

During the past 8 years, rifampicin-resistant TB incidence rates have consistently risen in Gabon, especially in the productive age group of the population. The extension of diagnostic capacity increases the total number diagnosed per year. Strengthening prevention and control programmes, actions should be initiated to reduce the burden of resistant tuberculosis. The national tuberculosis guideline should be revised for adding GeneXpert as first-line diagnostic tool to improve earlier (and hence cost-effective) case detection. We also recommend national-level implementation and cost-effective studies on decentralising drug-resistant tuberculosis treatment and home-based DOT. Moreover, further studies should be initiated to determine the transmission dynamics of multidrug-resistant strains using genotyping tools as well as studies aiming to investigate the risk factors for the development of MDR-TB in the population.

## Supplementary Information

Below is the link to the electronic supplementary material.

Supplementary file1 (DOCX 17 KB)

## Data Availability

Data are available from the corresponding author upon request.
