# Supplementary material for: Resistance patterns among drug-resistant tuberculosis patients and trends-over-time analysis of national surveillance data in Gabon, Central Africa
Source: Infection. 2022 Oct 28;51(3):697–704. doi: 10.1007/s15010-022-01941-5 (PMC9616411; doi:10.1007/s15010-022-01941-5)
Supplement: Supplementary file 1 — Supplementary file1 (DOCX 17 KB) [file 15010_2022_1941_MOESM1_ESM.docx]

| **Characteristic** | **[15-25], N = 55^1^** | **[25-35], N = 108^1^** | **[35-45], N = 67^1^** | **[45-55], N = 65^1^** | **[55-97], N = 39^1^** |
| --- | --- | --- | --- | --- | --- |
| **Patient category** |  |  |  |  |  |
| New patient | 16 (29%) | 35 (32%) | 18 (27%) | 24 (37%) | 10 (26%) |
| Previously treated for TB | 27 (49%) | 57 (53%) | 41 (61%) | 27 (42%) | 22 (56%) |
| Unknown | 12 (22%) | 16 (15%) | 8 (12%) | 14 (22%) | 7 (18%) |
| **SEX** |  |  |  |  |  |
| F | 30 (55%) | 56 (52%) | 25 (37%) | 25 (38%) | 14 (36%) |
| M | 25 (45%) | 52 (48%) | 42 (63%) | 40 (62%) | 25 (64%) |
| **HIV status** |  |  |  |  |  |
| Negative | 36 (65%) | 65 (60%) | 35 (52%) | 25 (38%) | 28 (72%) |
| Positive | 17 (31%) | 35 (32%) | 27 (40%) | 32 (49%) | 7 (18%) |
| Unknown | 2 (3.6%) | 8 (7.4%) | 5 (7.5%) | 8 (12%) | 4 (10%) |

**Supplementary File**

**Table S1.** Participants according to age category
